# Elevated Serum Levels of Macrophage Migration Inhibitory Factor Are Associated with Progressive Chronic Cardiomyopathy in Patients with Chagas Disease

**DOI:** 10.1371/journal.pone.0057181

**Published:** 2013-02-22

**Authors:** Romina A. Cutrullis, Patricia B. Petray, Edgardo Schapachnik, Rubén Sánchez, Miriam Postan, Mariela N. González, Valentina Martín, Ricardo S. Corral

**Affiliations:** 1 Servicio de Parasitología-Chagas, Hospital de Niños ‘Dr. Ricardo Gutiérrez’, Buenos Aires, Argentina; 2 Servicio de Cardiología, Hospital General de Agudos ‘Dr. Cosme Argerich’, Buenos Aires, Argentina; 3 Servicio de Cardiología, Hospital General de Agudos ‘Dr. José María Ramos Mejía’, Buenos Aires, Argentina; 4 Instituto Nacional de Parasitología ‘Dr. Mario Fatala Chabén’/ANLIS/Malbrán, Buenos Aires, Argentina; 5 Laboratorio de Inmunología, Centro de Salud y Medio Ambiente (CESyMA), Escuela de Ciencia y Tecnología (ECyT), Universidad Nacional de San Martín, Buenos Aires, Argentina; National Council of Sciences (CONICET), Argentina

## Abstract

Clinical symptoms of chronic Chagas disease occur in around 30% of the individuals infected with *Trypanosoma cruzi* and are characterized by heart inflammation and dysfunction. The pathogenesis of chronic chagasic cardiomyopathy (CCC) is not completely understood yet, partially because disease evolution depends on complex host-parasite interactions. Macrophage migration inhibitory factor (MIF) is a pleiotropic proinflammatory cytokine that promotes numerous pathophysiological processes. In the current study, we investigated the link between MIF and CCC progression.

Immunohistochemical analysis demonstrated MIF overexpression in the hearts from chronically *T. cruzi*-infected mice, particularly those showing intense inflammatory infiltration. We also found that MIF exogenously added to parasite-infected murine macrophage cultures is capable of enhancing the production of TNF-α and reactive oxygen species, both with pathogenic roles in CCC. Thus, the integrated action of MIF and other cytokines and chemokines may account for leukocyte influx to the infected myocardium, accompanied by enhanced local production of multiple inflammatory mediators. We further examined by ELISA the level of MIF in the sera from chronic indeterminate and cardiomyopathic chagasic patients, and healthy subjects. CCC patients displayed significantly higher MIF concentrations than those recorded in asymptomatic *T. cruzi*-infected and uninfected individuals. Interestingly, increased MIF levels were associated with severe progressive Chagas heart disease, in correlation with elevated serum concentration of high sensitivity C-reactive protein and also with several echocardiographic indicators of left ventricular dysfunction, one of the hallmarks of CCC. Our present findings represent the first evidence that enhanced MIF production is associated with progressive cardiac impairment in chronic human infection with *T. cruzi*, strengthening the relationship between inflammatory response and parasite-driven pathology. These observations contribute to unravel the elements involved in the pathogenesis of CCC and may also be helpful for the design of novel therapies aimed to control long-term morbidity in chagasic patients.

## Introduction

Chagas disease, caused by infection with the hemoflagellate protozoan *Trypanosoma cruzi*, is endemic in South and Central America, where an estimated 10 million patients are infected and another 100–120 million people are at risk of contracting the illness [1]. The disease is the most frequent worldwide cause of infectious cardiac pathology [2]. Approximately 30% of *T. cruzi*-infected patients develop heart dysfunction years or even decades after the initial infection. The pathogenesis of chronic chagasic cardiomyopathy (CCC) is not completely understood, partially because disease progression depends on complex host-parasite interactions. Four main pathogenetic mechanisms have been identified: direct *T. cruzi* damage to the myocardium, autoimmunity, dysautonomia, and microvascular disturbances [3]. CCC comprises a wide range of manifestations, including heart failure, arrhythmia, heart block, sudden death, thromboembolism, and stroke [4]. The main cardiac pathologic finding in Chagas disease patients is chronic progressive myocarditis [4]–[6], in which an important mononuclear infiltrate is accompanied by interstitial fibrosis and cardiomyocyte hypertrophy leading to dilated cardiomyopathy and ventricular systolic disorder that result in poor prognoses and high premature mortality rates [7]. It is widely accepted that the inflammatory infiltrate is the ultimate effector of myocardial damage and increased local expression of proinflammatory cytokines, chemokines, vascular mediators, HLA class I and II antigens, and adhesion molecules has been shown to contribute to *T. cruzi*-mediated heart dysfunction [8].

The proinflammatory cytokine macrophage migration inhibitory factor (MIF) participates in fundamental events of innate and adaptive immunity. MIF is present in many cell types and it is endowed with endocrine, enzymatic and receptor binding properties [9]–[11]. MIF binds and activates a multi-component receptor complex comprising CD74, CD44, and the chemokine receptors CXCR2 and CXCR4 [12], [13]. Moreover, MIF has been characterized as a physiologic counter-regulator of the anti-inflammatory activities of glucocorticoids [14]. In response to a variety of stimuli, MIF is released from preformed intracellular stores through an ABC transporter-dependent export pathway [15]. MIF promotes the production of a number of proinflammatory moieties, such as TNF-α, IL-6, IL-12 and reactive oxygen species (ROS), and it is required for normal leukocyte influx into inflamed tissues 9,16,17. Elevated serum levels of MIF were detected in many infectious and inflammatory diseases, such as rheumatoid arthrits [18], sepsis [19], vasculopathy [20], viral hepatitis [21], HIV infection [22] and malaria [23], indicating that MIF is implicated in pathogenesis. Additionally, MIF has been linked to the worsening of some pathologic conditions, and neutralization of this cytokine ameliorates the disease clinical course [24]–[27].

As a molecule that is detectable in the circulation and at the sites of inflammation, MIF might be a reliable marker of disease severity. The present study aimed to investigate the involvement of MIF in CCC. We observed that this inflammation-related factor is overexpressed in cardiomyocytes from chronically *T. cruzi*-infected mice and increased circulating MIF levels are associated with severe progressive heart disease in patients with CCC, in correlation with clinical features revealing impaired cardiac function. The integrated action of MIF and other cytokines and chemokines may account for leukocyte infiltration into the infected myocardium and also for enhanced local production of multiple inflammatory mediators.

## Materials and Methods

### Animals

Six- to eight-week-old female C3H/He mice were obtained from Centro Nacional de Energía Atómica (CNEA, Buenos Aires, Argentina) and maintained under standard conditions. Animals were housed in groups of five per cage and provided with food and water *ad libitum*. All experiments in this study were performed according to the National Research Counciĺs Guide for Animal Care and were approved by the Research and Teaching Committee and Bioethics Committee of Hospital de Niños ‘Dr. Ricardo Gutiérrez’.

### Parasites and Experimental Infection

Two established mouse models of chronic Chagas disease with or without evident cardiac pathology were used throughout our study. For experimental CCC, mice were infected i.p. with 10^6^ blood trypomastigotes of the Sylvio X10/4 clone of *T. cruzi* as described previously [28]. For the chronic/indeterminate (without apparent myocarditis) stage model, mice received 50 blood trypomastigotes of the Tulahuén strain of *T. cruzi* as reported [29]. Infected animals and uninfected age-matched controls were ether anesthetized and euthanized by cervical dislocation at 120 days p.i, making all efforts to minimize suffering of mice. Hearts were removed, sectioned and stored under specific conditions for diverse assays.

### Immunohistochemical Studies

Immunohistochemical analysis was performed on formalin-fixed, paraffin-embedded cardiac muscle specimens from infected and uninfected mice. Five- µm sections were cut onto coated slides and were deparaffinized using routine techniques. After blocking endogenous peroxidase with 3% hydrogen peroxide and nonspecific binding sites with 2% bovine serum albumin, rabbit anti-mouse MIF polyclonal antibodies (Zymed Laboratories, San Francisco, CA, USA) were applied to the sections. As secondary antibody, we used biotynilated swine anti-rabbit IgG polyclonal antibodies (Dako, Glostrup, Denmark). The reaction product was revealed by streptavidin-horseradish peroxidase complex with diaminobenzidine tetrahydrochloride and hydrogen peroxide chromogen substrate (Dako LSAB® + System-HRP). The sections were then counterstained with Mayeŕs hematoxylin and periodic acid-Schiff. Omission of the primary antibody and use of isotype-matched control antibodies served as controls.

### Flow Cytometry

For the analysis of the leukocyte infiltrate, hearts from 20 infected mice (120 days p.i.) with CCC were enzymaticaly digested at 37°C with 200 FALGPA U/ml collagenase type IV from *Clostridium histolyticum* and 200 FALGPA U/ml hyaluronidase type IV-S (Sigma-Aldrich, St. Louis, MO, USA) to isolate inflammatory cells. The mononuclear cell fraction was separated by centrifugation on Histopaque 1083 (Sigma-Aldrich) [30] and washed twice with PBS. Cell viability was assessed by Trypan blue dye exclusion. The cells were suspended in PBS with 10% fetal calf serum (FCS) and incubated for 30 min at 4°C with 10 µl of 2.4G2 rat anti-mouse FcγRII/RIII (a kind gift of G. Mirkin, University of Buenos Aires) to avoid nonspecific staining. After rinse, labeled rat anti-mouse CD11b/MAC-1- PerCP-Cy 5.5 (1∶200 dilution), CD3-FITC (1∶100), CD4-PE (1∶200) and CD8-Alexa Fluor 647 (1∶200) antibodies (BD Biosciences-Pharmingen, San José, CA, USA) were added to the cell suspension at a final volume of 100 µl, incubated in the darkness at 2–8°C for 30 min and fixed with fresh 1% *p*-formaldehyde at room temperature. Samples (at least 5×10^4^ cells) were acquired in a Partec Past III flow cytometer and selected in the monocyte population gate according to their forward scatter versus side scatter features. Data were analyzed using Cyflogic 1.2.1 software.

### TNF-α Measurement in the Supernatant of *Trypanosoma cruzi*-Infected J774 Cells

The J774 macrophage cell line was maintained by weekly passages in complete RPMI 1640 medium containing 10% FCS, 2 mM L-glutamine, 200 U/ml penicillin and 200 µg/ml streptomycin.. Murine macrophages (5×10^5^/well) were seeded in 12-well tissue culture plates and the adherence was allowed for 24 h. Thereafter, adherent cells were infected for 24 h with culture trypomastigote forms of *T. cruzi* (Tulahuén strain) at a 10∶1 parasite/cell ratio, in the presence or in the absence of recombinant MIF (1 µg/ml, R&D Systems, Minneapolis, MN, USA). TNF-α production was quantified in uninfected and parasite-infected J774 cell supernatants using a sandwich ELISA (OptEIA™ Mouse TNF, BD Biosciences-Pharmingen) according to the manufacturer's instructions. Supplied standards were used to generate the standard curve. The assays sensitivity was 15 pg/ml.

### Quantification of Intracellular ROS Levels

ROS generation was measured by the DCFH-DA (2′,7′-dichlorodihydrofluorescein diacetate, Sigma-Aldrich) fluorescence method. Briefly, J774 macrophages (10^6^) were washed, suspended in 1 ml of PBS and incubated with 10 µM DCFH-DA for 30 min at 37°C. The cells were then infected for 24 h with *T. cruzi* trypomastigotes at a 10∶1 parasite/cell ratio, in the presence or in the absence of recombinant MIF (1 µg/ml). Uninfected cells were included as a control. Macrophages were then fixed with 4% *p*-formaldehyde for 15 min. Cells were harvested, repeatedly washed and analyzed by flow cytometry at a wavelength of 488 nm. Data analysis was performed using Cyflogic 1.2.1 software.

### Study Population

Studies described herein received ethics clearance from the Institutional Review Boards of the Hospital General de Agudos Dr. Cosme Argerich -CA- and Hospital General de Agudos José María Ramos Mejía -RM- (Buenos Aires, Argentina). Fully informed written consent was obtained from all patients and noninfected individuals. The human experimentation guidelines of the World Health Organization and the Declaration of Helsinki were followed in the conduct of our experiments.

Subjects were recruited at the Cardiology units of CA and RM public hospitals. Most participants were Argentinean (70.7%); the rest were from Paraguay (17.1%) and Bolivia (12.2%). *T. cruzi* infection was determined by a combination of assays [indirect hemagglutination (Polychaco SAIC, Buenos Aires), particle agglutination (Fujirebio Inc., Tokyo, Japan), and ELISA (Wiener Lab, Rosario, Santa Fe, Argentina)]. Subjects positive on at least two of these tests were considered to be infected. Chronic chagasic patients were evaluated clinically and grouped according to the Kuschnir grading system [31]. Group 0 (G0, *n* = 14; age range = 38–59 years; mean age = 51.9 years) included seropositive individuals exhibiting a normal electrocardiogram (ECG), echodopplercardiography (ECHO) and chest radiographic findings, and group 3 (G3, *n* = 12; age range = 40–65 years; mean age = 56.3 years) seropositive patients with ECG and ECHO abnormalities, conduction defects, heart enlargement, and clinical or radiological evidence of heart failure. The G0 and G3 classification corresponded to the indeterminate form of Chagas disease and severe CCC, respectively. The noninfected control group (*n* = 15; age range = 30–64 years; mean age = 47.3 years) consisted of healthy individuals who were serologically negative for *T. cruzi* and who had not had heart failure (Table 1). Infected and control subjects with hypertension, congenital heart disease, hypercholesterolemia, vascular or ischemic disease, cancer, clinical evidence of any infectious disease, arthritis, diabetes, allergy, or inflammatory/autoimmune disorder were excluded from the study.

**Table 1:** Age and sex distribution, and echocardiographic and electrocardiographic parameters in chronic chagasic patients and in noninfected individuals.

	NoninfectedIndividuals(*n* = 15)	IndeterminateChagas patients(*n* = 14)	CardiomyopathicChagas patients(*n* = 12)	P
**Characteristics**				
Age (years)	47.3±1.4	51.9±1.7	56.3±6.8	<0.05
Gender (F/M)	8/7	10/4	1/11	<0.0001
**Echocardiography**				
LVEDD (cm)	4.4±0.2	4.4±0.1	6.4±0.3	<0.001
LVESD (cm)	2.5±0.3	2.5±0.1	5.2±0.3	<0.001
LVFS (%)	38.0±4.0	44.0±1.8	17.0±3.4	<0.001
IS (cm)	0.9±0.2	0.9±0.1	1.2±0.1	>0.05
PW (cm)	0.9±0.1	0.8±0.1	0.8±0.1	>0.05
LAD (cm)	3.4±0.2	3.3±0.1	4.9±0.3	<0.001
AD (cm)	2.7±0.1	2.7±0.1	3.2±0.1	<0.001
**Electrocardiography**				
Abnormal outcome				
(% of patients)	0.0	0.0	100.0	

**LVEDD**, left ventricular end-diastolic diameter; **LVESD**, left ventricular end-systolic diameter; **LVFS**, fractional shortening; **IS**, interventricular septum; **PW**, posterior wall; **LAD**, left atrial diameter; **AD**, aortic diameter. Parameters and age are expressed as mean ± SEM values. *P* values for the comparison of group means (cardiomyophatic Chagas patients *vs* indeterminate Chagas patients/noninfected individuals) are also depicted. The nonparametric Kruskall-Wallis test was used to compare age means and Fisheŕs exact test for gender proportion. One-way ANOVA test was conducted for the comparative analysis of the echocardiographic parameters.

### Measurement of Cytokine and C-Reactive Protein Levels

Serum levels of human MIF and TNF-α were quantified by double sandwich ELISA (DuoSet® ELISA Development System, R&D Systems, and ChemiKine™, EMD Millipore, Billerica, MA, USA, respectively) according to manufacturers' instructions. Supplied standards were used to generate the standard curve for each cytokine. The assay sensitivity was 125.0 pg/ml and 4.8 pg/ml for MIF and TNF-α, respectively.

High sensitivity C-reactive protein (HS-CRP) was measured in human serum specimens by using latex-enhanced immunonephelometry (CardioPhase HS-CRP, Siemens Medical Solutions Diagnostics, Deerfield, IL, USA) with a lower detection limit of 0.4 mg/L and an intra-assay coefficient of variation of 1.2%.

### Statistical Analyses

Data analysis was carried out using Prism 5.0 software (GraphPad Software Inc., San Diego, CA, USA). Data are expressed as mean value ± standard error of the mean (SEM). ANOVA, Bonferronís multiple comparison, Kruskal-Wallis and Fisheŕs tests were applied to compare different groups. Pearsońs correlation was used to analyze the covariation between variables. Multiple regression analysis was performed using Statistica 8.0 software (StatSoft Inc., Tulsa, OK, USA). Differences were considered statistically significant when *P*<0.05.

## Results

### Myocardial MIF Expression and Leukocyte Influx in *Trypanosoma cruzi*-Infected Mice

**Figure 1:**
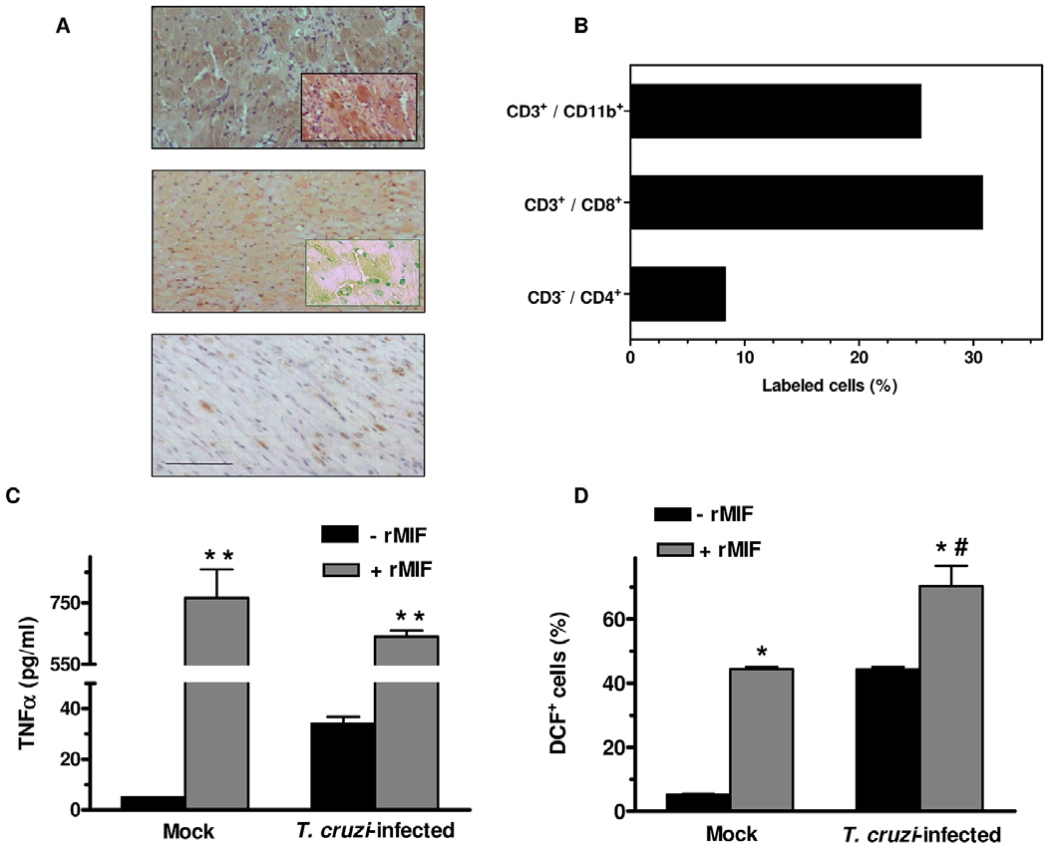
MIF is overexpressed in the heart of chronic chagasic mice and potentiates production of TNF-α and ROS by *Trypanosoma cruzi*-infected macrophages. C3H/He mice (*n* = 5) were infected intraperitoneally with 10^6^ blood trypomastigotes of the Sylvio X10/4 clone of *T. cruzi* (upper panel) or with 50 blood trypomastigotes of the Tulahuén strain of the parasite (central panel). Hearts from infected animals and uninfected controls (lower panel) were removed at 120 days p.i. (A) Immunohistochemical analysis was performed on cardiac muscle sections using murine MIF polyclonal antibodies. Microphotographs of myocardial tissues show a representative experiment of three performed. Bar = 50 µm. (B) MIF overexpression in the heart was closely accompanied by intense inflammatory cell infiltration. The leukocyte population invading the myocardium was isolated from 20 *T. cruzi*-infected mice at 120 days p.i. and digested with collagenase and hyaluronidase. The mononuclear cell fraction was incubated with anti-mouse CD11b/MAC-1- PerCP-Cy 5.5, CD3-FITC, CD4-PE and CD8-Alexa Fluor 647 antibodies. The labeled cells (at least 5×10^4^) were fixed with 1% *p*-formaldehyde and analyzed by flow cytometry. (C), (D) Effect of exogenous MIF on *T. cruzi*-induced release of TNF-α and ROS in murine J774 macrophages. Adherent cells were infected for 24 h with culture trypomastigotes of *T. cruzi* (Tulahuén strain) at a 10∶1 parasite/cell ratio, in the presence (+ rMIF) or in the absence (− rMIF) of recombinant mouse MIF (1 µg/ml). TNF-α production was quantified in uninfected (Mock) and parasite-infected cell supernatants using a sandwich ELISA (C). For ROS measurement. J774 macrophages (10^6^) were incubated with 10 µM DCFH-DA (2′,7′-dichlorodihydrofluorescein diacetate) for 30 min and then infected for 24 h with *T. cruzi* trypomastigotes with or without addition of rMIF, as described above. Uninfected (Mock) and infected cells were then fixed and analyzed by flow cytometry (D). Data are the means ± S E M of three independent experiments, each performed in triplicate. ^*^ *P*<0.05 and ^**^ *P*<0.01 versus cells not pre-treated with MIF; #*P*<0.05 between infected and uninfected MIF-stimulated cells.

*T. cruzi*-driven induction of MIF in the hearts from chronically infected mice was investigated by immunohistochemistry. Using a murine model of CCC, we found that MIF protein expression was strongly upregulated in the myocardial inflammatory lesions at a time of infection (120 days p.i.) when heart parasitism and marked leukocyte infiltration are both present [28]. Enhanced reactivity for the cytokine was mostly localized in the cardiomyocytes (Figure 1A, upper panel), even though a few MIF-expressing infiltrating cells could be visualized as well. MIF was also identified, albeit to a much lesser degree, in cardiac tissues from mice under indeterminate stage of chronic *T. cruzi* infection (Figure 1A, central panel). Only a very weak basal expression of MIF could be observed in specimens from uninfected animals (Figure 1A, lower panel). As MIF has been originally found to inhibit the random migration of monocytes/macrophages [32], we asked whether the local expression of this proinflammatory cytokine could lead to mononuclear cell recruitment to and accumulation in cardiac tissues from CCC mice. The leukocyte population invading the heart of *T. cruzi*-infected mice at 120 days p.i. was isolated and subjected to FACS analysis (Figure 1B). In agreement with previous reports [33], [34], we observed that the main subset was constituted by CD3^+^ CD8^+^ T cells. Of note, 25.4% of mononuclear cells infiltrating cardiac tissues belonged to the monocytic lineage (CD3^−^, CD11b^+^). Taken together, our results show that this intense leukocyte infiltration is closely accompanied by myocardial MIF overexpression. Thus, increased MIF production by cardiac myocytes might be contributing to the migration and homing of inflammatory cells in the heart of mice chronically infected with the parasite.

### MIF Potentiates *Trypanosoma cruzi*-Induced Production of TNF-α and ROS in Macrophages

MIF has been shown to promote the synthesis of several mediators of inflammation, including TNF-α and ROS [9], [17]. Therefore, we next examined whether MIF was capable of enhancing the well-known ability of *T. cruzi* to induce ROS generation and TNF-α secretion by macrophages [35], [36]. Intracellular ROS formation and cytokine levels in the supernatant from uninfected cultures were practically negligible. After 24 h of parasite infection, murine J774 cells produced detectable amounts of TNF-α (34±5 pg/ml) that were strikingly raised (640±35 pg/ml, *P*<0.001) by adding recombinant mouse MIF (1 µg/ml) (Figure 1C). In addition, MIF-treated macrophages presented significantly increased ROS levels compared with untreated infected cells (70±7 vs. 44±1% of fluorescent cells, respectively; *P*<0.05) (Figure 1D). These findings indicate that, in *T. cruzi*-infected phagocytes, MIF stimulates the production of ROS and TNF-α, both relevant pathogenic mediators of CCC [37], [38].

### Circulating Levels of MIF and TNF-α in Patients with Indeterminate or Cardiomyopathic Chronic Chagas Disease

**Figure 2:**
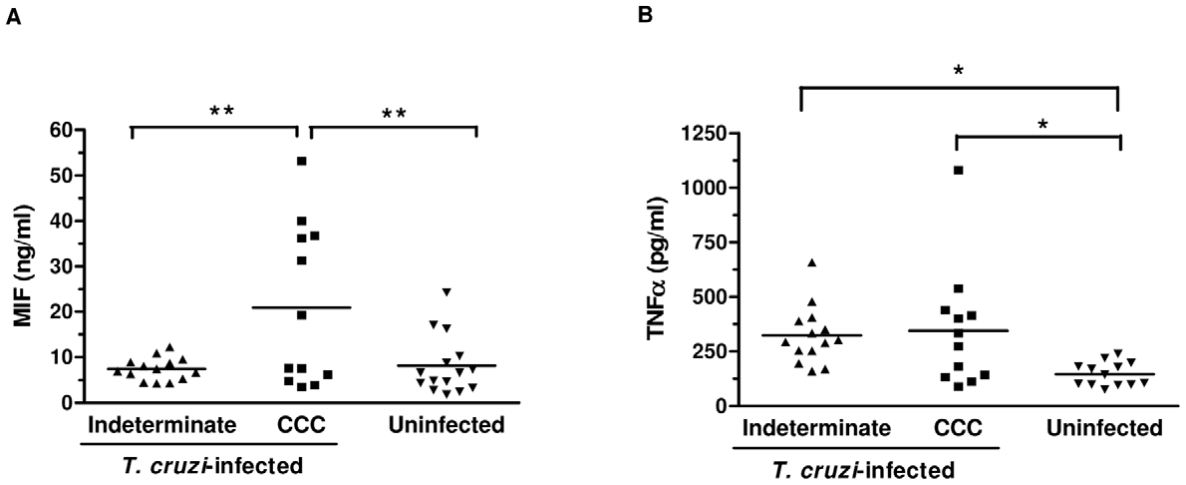
Concentrations of MIF and TNF-α in serum samples collected from patients chronically infected with *Trypanosoma cruzi*. Infected patients without evident cardiac involvement (▴indeterminate, *n* = 14) or with Chagas cardiomyopathy (▪ CCC, *n* = 12), and uninfected controls (▾; *n* = 15) were studied. MIF (A) and TNF-α (B) levels were measured by ELISA in three independent experiments, and individual results for each patient are given. The horizontal lines represent mean values in each group. ^*^ *P*<0.05; ^**^ *P*<0.01.

Other groups have shown that CCC is frequently accompanied by increased concentration of proinflammatory cytokines in the circulation of chronically infected individuals [38], [39]. In our series, CCC patients displayed higher serum levels of MIF detected by ELISA than those recorded in asymptomatic *T. cruzi*-infected and noninfected groups (20.9±5.1 vs. 7.4±0.6 and 8.1±1.6 ng/ml, respectively) (Figure 2A). Also, both CCC patients and long-term infected subjects without evidence of cardiomyopathy showed enhanced concentration of TNF-α in blood compared to healthy people (324.6±41.4 and 344.3±79.4 vs. 146.9±14.8 pg/ml, respectively) (Figure 2B). Nonetheless, no significant correlation between both cytokine levels could be demonstrated (unpublished observation).

As shown in Table 1, age and gender were unequally distributed in our study groups. The average age of CCC patients was significantly different (*P*<0.05) from those of noninfected controls and indeterminate Chagas patients. Distribution according to sex was also unbalanced, with a extremely marked (*P*<0.0001) decrease in the proportion of women within the group of infected subjects with severe cardiomyopathy. In addition, highly significant (*P*<0.001) alterations in several ECHO parameters were found in CCC patients (Table 1). Consequently, multiple regression analysis between MIF values and age/sex/form of chronic Chagas disease was further conducted in order to verify the influence of independent variables on the levels of proinflammatory factor. In our study population, there was no significant correlation between circulating MIF concentration and either age, or sex, or the indeterminate form of Chagas disease (*P* = 0.106, 0.223 and 0.556, respectively). Conversely, statistical analysis confirmed the hypothesis that MIF values significantly (*P* = 0.006) correlate to advanced CCC.

**Figure 3:**
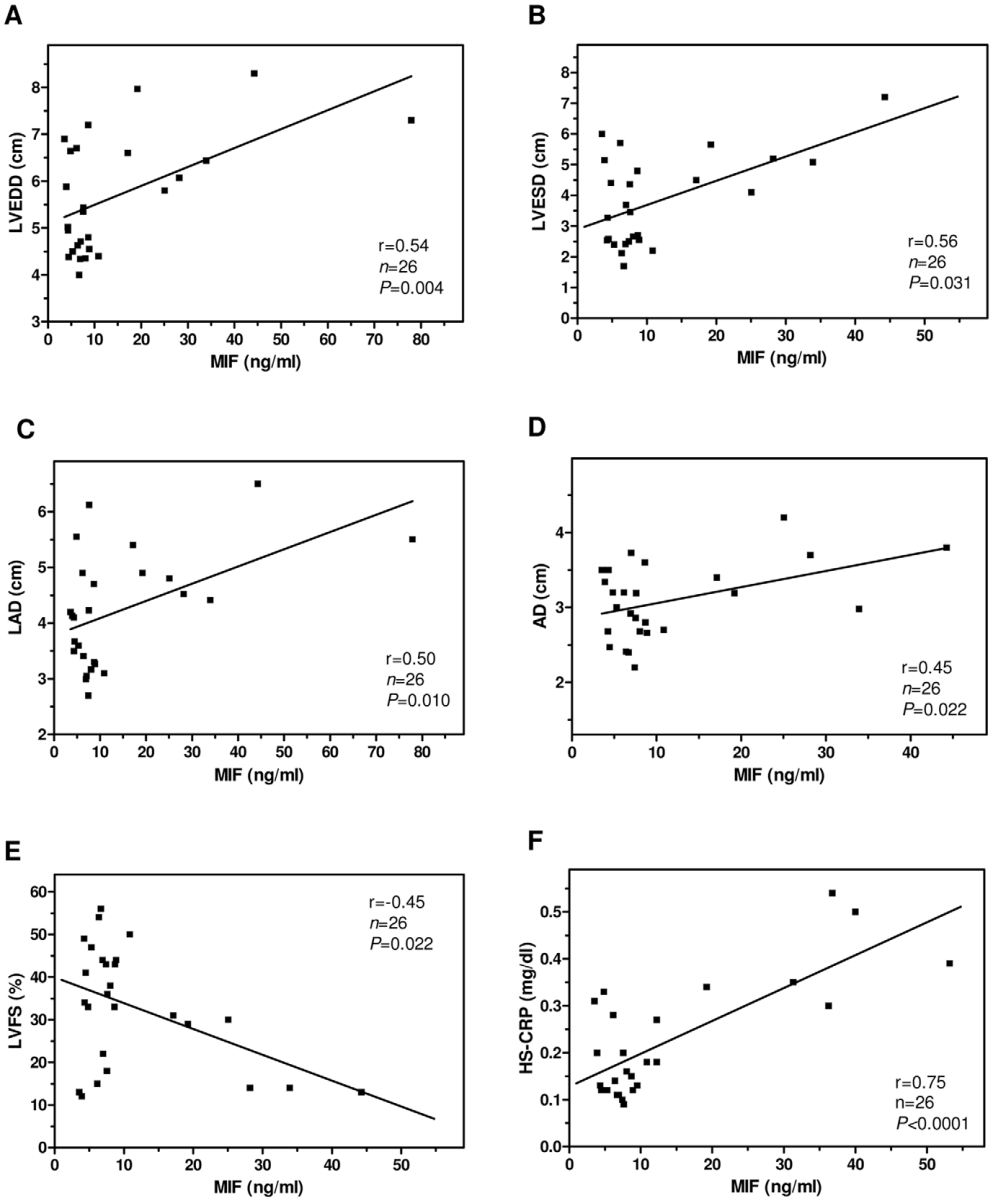
Correlation between serum MIF level and parameters of cardiac dysfunction in chagasic patients. (A) LV end-diastolic diameter (LVEDD), (B) LV end-systolic diameter (LVESD), (C) left atrial diameter (LAD), (D) aortic diameter (AD), (E) LV per cent fractional shortening (LVFS), and (F) serum concentration of MIF and high sensitivity C-reactive protein (HS-CRP) were analyzed in chagasic (both chronic indeterminate and cardiomyopathic) patients (*n* = 26). MIF concentration was measured by ELISA; LVEDD, LVESD, LAD, AD and LVFS were determined by echodopplercardiography; HS-CRP level was quantified by immunonephelometry. Individual results are shown for each patient; the line represents the linear regression for each comparison. Alpha level was adjusted to 0.01. The correlation coefficient (r) and *P* values for each association are indicated.

In addition, serum MIF values from chagasic patients correlated with ECHO indicators of left ventricular (LV) dysfunction, one of the hallmarks of CCC [3] (Figure 3). For instance, MIF concentrations presented strong correlation with the increases of LV end-diastolic diameter (LVEDD, r = 0.54, *P* = 0.004), LV end-systolic diameter (LVESD, r = 0.56, *P* = 0.031), left atrial diameter (LAD, r = 0.50, *P* = 0.010), and aortic diameter (AD, r = 0.45, *P* = 0.022), as well as with the decline of LV per cent fractional shortening (LVFS, r = −0.45, *P* = 0.022) (Figure 3, A–E). Furthermore, circulating MIF levels in *T. cruzi*-infected individuals showed a very significantly positive correlation (r = 0.75, *P*<0.0001; Figure 3F) with the amount of HS-CRP, a serum biomarker linked to serious heart pathology in Chagas disease [40]. Collectively, our findings suggest that augmented MIF production is associated with progressive cardiac impairment in patients suffering from CCC.

## Discussion

Recently, MIF has been considered a critical mediator of the early immune responses to different protozoan infections (reviewed in [41]). As for other cytokines that play a dual role during the course of infection [42], MIF may be additionally involved in the pathophysiology of parasitic diseases. In this regard, augmented MIF circulating levels detected in *Plasmodium*-infected patients have been associated with anemia and lethal cerebral malaria [23], [43]. Likewise, MIF has been shown to participate in *Toxoplasma gondii*-induced pathology following oral infection in mice, whereas bloodstream MIF was found to positively correlate with lipopolysaccharide (LPS) plasma levels and chronic immune imbalance in active visceral leishmaniasis patients [44], [45].

In Chagas disease, early MIF induction in target tissues from infected hosts has been reported as a crucial step for resistance to acute *T. cruzi* infection [46]–[48]. On the other hand, MIF and TNF-α are important for the lethal synergism between *T. cruzi* infection and LPS-induced shock [49]. However, the role of MIF in the establishment of CCC remains to be elucidated. We herein found that chronically infected mice display progressive MIF overexpression in cardiac myocytes which is likely to contribute to parasite-triggered inflammatory cardiomyopathy. Previous reports have revealed that the myocardium is itself a significant source of MIF [47], [50] and the expression of this cytokine in cardiomyocytes is upregulated upon heart injury [51].

MIF has been described as a key mediator of macrophage/monocyte influx and promoter of T cell migration and arrest in inflamed tissues as well [13], [52]. Taking into account its chemoattractant activity, it seems conceivable that elevated local production of this inflammatory agent would be at least partially responsible for leukocyte trafficking to the heart throughout *T. cruzi* infection. In our animal model of CCC, the highest level of local MIF expression was demonstrated in heart tissues exhibiting an important inflammatory infiltration. As expected, the phenotypic analysis of infiltrates isolated from the myocardium of CCC mice showed a dominance of CD8^+^ T lymphocytes accompanied by a significant CD11b-expressing leukocyte subset. These CD11b^+^ cells were most likely monocytes/macrophages, since infiltrating polymorphonuclear cells are rarely seen at this stage of experimental *T. cruzi* infection [53]. Coincidently, using a different mouse model of CCC, Talvani et al. [53] observed a gradual increase in CD11b^+^ mononuclear cells, ranging from 20% up to 50% of the inflammatory infiltrate at 90 and 120 days p.i., respectively. Nevertheless, myeloid-derived suppressor cells (MDSCs), with CD11b^+^ Gr1^+^ cell phenotype, could also be present in cardiac tissue of *T. cruzi*-infected mice [54]. Interestingly, MIF has been shown to increase the prevalence and promote differentiation of a highly immunosuppressive subpopulation of MDSCs [55].

Further, MIF could favor heart injury by inducing the secretion of proinflammatory cytokines and ROS from infiltrating leukocytes. In this regard, MIF appears to potentiate the release of cytotoxic TNF-α from *T. cruzi*-harboring macrophages, akin to the effect caused by IL-3 on parasite-infected peripheral blood cells [56]. It is widely accepted that macrophages that constitute a considerable portion of the inflammatory infiltrate in the heart are a major local source of TNF-α [57]. Overexpression of this cytokine was observed in myocardial lesions and has been linked to progressive cardiac disease caused by *T. cruzi* infection [58], [59]. Among patients with Chagas' cardiomyopathy who also have overt heart failure, particular TNF-α genotypes have been associated with a significantly shortened survival [60]. We also found that MIF is capable of enhancing ROS generation by parasite-infected macrophages. A mechanism through which MIF can promote ROS production has been described recently [17]. Upon receptor binding, the MIF-CD74 complex is internalized through endocytosis, resulting in activation of NADPH oxidase and generation of ROS within early endosome. The occurence of such process in our experimental model deserves further investigation. There is growing evidence to suggest that chagasic myocardia are exposed to sustained oxidative stress-dependent injuries that may contribute to disease progression [37]. The level of oxidative damage biomarkers in the myocardium consistently increases with the severity of pathology, and ROS-induced, cardiac-oxidized antigens have recently been postulated as molecular determinants for pathogenesis during Chagas disease [61]. The extracellular release of ROS, produced either by the myocardium itself or by heart-infiltrating inflammatory leukocytes, may further trigger pathologic signaling on adjacent cells [62]. ROS have been shown to cause protein carbonylation, membrane lipoperoxidation and oxidative DNA modifications in cardiac tissues leading to oxidant/anti-oxidant imbalance and mitochondrial dysfunction during chagasic cardiomyopathy development [63]–[65]. Remarkably, cytokines such as IL-1β and TNF-α which are induced by MIF have been implicated in the induction of autophagy via ROS [17], thereby contributing to pathogenesis and progression of infectious myocarditis [66]. To sum up, all the pieces of evidence support the hypothesis that MIF could be mediating myocardial inflammatory damage in chronically *T. cruzi*-infected mice through the progressive recruitment and sustained activation of mononuclear cells. However, to fully validate the role of MIF in chagasic infection a complete inhibition of its expression in infected cells (cardiomyocytes, macrophages and fibroblasts) should be achieved [67].

Augmented circulating proinflammatory cytokine level is a regular feature of symptomatic *T. cruzi* infection in humans [38], [39]. In our population of chronically infected patients, serum concentrations of MIF and TNF-α were higher than those exhibited by uninfected individuals. Positive correlation between both cytokines could not be established, possibly due to distinct regulatory mechanisms for producing each immune mediator during the course of infection. The mean serum concentration of MIF in the CCC group was 20.9 ng/ml, which is comparable with the level reported previously in patients with pulmonary tuberculosis (mean of 19.8 ng/ml [68]). Remarkably, our findings show for the first time that chronic chagasic patients with evident cardiac involvement differentially display increased systemic MIF levels correlating with ECHO (LVEDD, LVESD, LVFS, LAD and AD) and laboratory (circulating HS-CRP) indicators of advanced heart damage. LV dilatation is a major symptom in chronic *T. cruzi*-infected patients with cardiac impairment and poor prognosis [3], [6], and significant HS-CRP elevation has been detected in the most serious clinical forms of CCC [40]. Interestingly, recent studies demonstrated that coxsackievirus B3-induced myocarditis as well as human diabetes-linked LV dysfunction correlate with increased MIF concentration in the bloodstream [69], [70]. Also, high levels of MIF showed good correlation with CRP in the sera from patients with rheumatoid arthritis [71].

In conclusion, our results suggest that enhanced MIF production is associated with severe progressive cardiac dysfunction in patients with chronic Chagas disease, strengthening the relationship between pathogenic mechanisms of inflammatory response and the intensity of cardiac failure caused by prolonged infection. Even though MIF elevation is not exclusively related to CCC, it may be complementary to other inflammatory markers for evolution of Chagas disease to more advanced stages of cardiovascular impairment. Therefore, the measurement of circulating MIF levels could serve as an additional tool for identifying chronic chagasic patients with serious heart pathology who may benefit from further investigation and treatment. The characterization of the different cytokines present in cardiac tissue and in the bloodstream during *T. cruzi* infection and their correlation with the degree of myocardial compromise can be helpful for unraveling the elements involved in the pathogenesis of CCC and also for the design of novel therapies aimed to control chronic morbidity in chagasic patients.
